# Globally competent teachers: English as a second language teachers’ perceptions on global competence in English lessons

**DOI:** 10.3389/fpsyg.2022.925160

**Published:** 2022-08-03

**Authors:** Nur Syafiqah Yaccob, Melor Md. Yunus, Harwati Hashim

**Affiliations:** ^1^Sekolah Menengah Kebangsaan Dato’ Abdul Rahman Ya’kub, Melaka, Malaysia; ^2^Faculty of Education, Universiti Kebangsaan Malaysia, Bangi, Malaysia

**Keywords:** English as a second language, global competence, globally competent ESL teachers, teachers’ perceptions, teaching and learning of English

## Abstract

Due to the implementation of global education and global citizenship education in the 21st century, more focus is given to developing teachers’ global competence in English language teaching. This study aims to examine the perceptions of English as a Second Language (ESL) teachers of (1) global competence integration in English teaching and (2) the professional development programs organized by the Ministry of Education (MOE) for their global competence development. A web-survey questionnaire was distributed to 172 Malaysian ESL teachers based on selected criteria. The data collected were analyzed descriptively. The main findings indicated that most ESL teachers showed positive perceptions regarding the importance of global competence and integration in English lessons. Although 83.1% of the 172 participants agreed to have attended 10 or more programs related to developing global competence, the descriptive analysis found the ESL teachers’ moderate knowledge and understanding of what constitutes global competence. In contrast, a high agreement was found regarding their perceptions of the importance of global competence in ESL lessons. Also, most respondents revealed the lack of support from the MOE through professional development programs specifically structured to develop ESL teachers’ global competence. The findings served as a template for a larger-scale study that focuses on implementing global competence in the local non-western context.

## Introduction

The English language has become an essential language worldwide. It is the *lingua franca* in many fields, including education, politics, economics, social sciences, and technologies (Bullah and Yunus, 2019). It holds its position as a widely-used language of instructions. For non-native English speakers, being competent in several languages, including English, is a prerequisite to adapt and be connected in today’s multicultural and mobile society (Heinzmann et al., 2015). Furthermore, Halim et al. (2020) described competent English language users as assets for societal and country development. Since English is the language of knowledge in most schools, it becomes the medium of instruction and information content delivery. Malaysian schools have long used English as the language of communication as it is learned as a second language (L2). However, some scholars expressed concerns about the deteriorating Malaysian students’ English proficiency and literacy (Halim et al., 2020), and the Ministry of Education (MOE) in Malaysia continuously emphasizes the importance of competency in English acquisition among students (Bullah and Yunus, 2019).

Over the last decade, teachers’ responsibilities have expanded to educating students about a complex and interconnected society (Tichnor-Wagner et al., 2019). Therefore, education today requires a new dimension of teachers’ professional development that correlates with global competencies (Orazbayeva, 2016). Likewise, continuous professional development for teachers is to develop proficiencies to meet changing professional demands by regularly involved in various updated programs (Sukri et al., 2017; Vadivel et al., 2021). From a language student’s standpoint, students need to be taught English by highly trained language teachers who are constantly ready to adapt to changes in the educational system throughout the world. They must be knowledgeable about global concerns and possess the required abilities to be competent English teachers in various settings (Orazbayeva, 2016; Salzer and Roczen, 2018; Diveki, 2020; Yaccob et al., 2021).

Language education aims to prepare students for effective interaction and collaboration since English constantly evolves, as all languages do (Othman and Lotfie, 2019). Byram and Wagner (2018) identified language teaching as preparation for students to be proficient users of the target language. The authors added that students need to learn to interact with people from various cultural backgrounds with the necessary skills, attitudes, and knowledge. The above mentioned students’ needs are among the global competence elements regarding cross- cultural interaction (Tichnor-Wagner et al., 2019; OECD, 2020). Thus, English, precisely English as a Second Language (ESL) teachers, ought to create lessons that will develop students’ global competence. This is why globally competent ESL teachers have the knowledge, skills, and dispositions to do so (Yaccob et al., 2021). According to Salzer and Roczen (2018) and Mansilla and Wilson (2020), globally competent ESL teachers can create meaningful and comprehensive English lessons for students. They can effectively work in diverse classrooms and school environments (Salzer and Roczen, 2018; Diveki, 2020; Yaccob et al., 2021). It is significant in the Malaysian ESL context since Malaysia is considered one of the most heterogeneous countries worldwide (Abdullah and Abdullah, 2018).

### Literature review

Many researchers have researched teachers’ global competence development and implementation in lessons [e.g., Kerkhoff et al. (2019); Sinagatullin (2019); Tichnor-Wagner et al. (2019)]. However, most are from western viewpoints and settings. Hence, it is deemed unfit to generalize those past studies into the Malaysian ESL context. ESL teachers’ teaching of global competence in English lessons influences students’ language development and understanding of world knowledge. Some students may not engage with people and cultures outside their classroom walls (Tichnor-Wagner et al., 2019). Besides, students’ lack of preparation to interact with diverse cultures, ideas, and opinions in the current global society withheld their global competence development (Fox, 2019). When students were not exposed to global knowledge, such as intercultural knowledge, they had a limited understanding of cultural differences (Popescu and Iordachescu, 2015). Consequently, global competence in ESL lessons will highly influence the development of students’ global competence to think of how a classroom should become the lens to see the world.

The practice of educating for global competence responds to globalization demands (Tamerat, 2020). Global competence, as defined by OECD (2020), refers to the abilities of individuals/students to:

i. examine local, global, and cultural issues,
ii. understand and appreciate others’ perspectives and worldviews,
iii. engage in cross-cultural interactions and collaborations.
iv. take action for sustainable development and to protect community wellbeing.

However, global competence cannot be developed in students over a short period of time (Tichnor-Wagner et al., 2019). It requires ESL teachers’ efforts to incorporate it into every language lesson. The Programme for International Students Assessment (PISA) by the Organisation for Economic Co-Operation and Development (OECD) has already started to measure students’ global competence through assessments (Diveki, 2020). The global competence test reported by Education GPS, OECD (2021) stated that Malaysian students had the lowest awareness level of global issues with the PISA index of-0.41 and ranked 60 among 64 PISA-participating countries. Besides, according to Avvisati et al. (2019), the students showed lower than the average performance by scoring 54% (Level 2) OECD score, compared to the OECD average of 77%. It reflects the lack of global competence among students. The authors added that the results proved the students’ weakness in reading English materials, such as understanding the meaning and main idea in OECD reading literacy’ texts.

Therefore, getting minimum results in PISA reflects their deficiency of global awareness in real-life. A study by Majzub et al. (2017) on students’ global readiness indicated that ICT usage and problem-solving dimensions had a high degree of global preparedness, whereas students’ communication and environmental awareness were at a moderate level. The authors deduced that students might not be aware of disasters in other countries, such as the earthquakes in Japan, due to their moderate level of global awareness. It highlights the lack of global competence integration into some English classrooms.

Similarly, ESL teachers’ global competence is vitally significant when textbooks contain insufficient global and local knowledge. Although cultural references are prevalent in textbooks and media, they are often restricted to the “American,” “British,” and “European” cultures, ignoring other cultures around the world in which English is not a native language, such as Asian and the Middle Eastern (Galante, 2015; Johar and Abdul, 2019). Meaningful language learning refers to contextual learning that includes knowledge of the interconnected world. ESL teachers help students better examine and deal with global issues through active and authentic language learning (Fox, 2019). Another benefit is instilling ethical sensitivity among students (Stankovska et al., 2019). The authors explained that having ethical sensitivity enables students to think critically about social situations, analyze real-life issues, be creative and innovative in identifying necessary solutions, and behave ethically in society (Stankovska et al., 2019). Therefore, professional development programs for teachers and ESL teachers must include methods to integrate global competence in lessons (Yaccob et al., 2022). For teachers to become globally competent as required by the current global education, they need a suitable and structured program across content areas as a guideline (Tichnor-Wagner et al., 2019). In supporting English teachers’ professional quality, Liu et al. (2021) suggested the construction of professional learning communities that maintain and promote their professional development.

This study intends to bridge the gap in the area by examining the perceptions and opinions of ESL teachers on global competence in English lessons. It also identifies the perceptions of ESL teachers on professional development programs by the MOE in developing global competence among English teachers. This study helps inform policymakers of teachers’ perceptions and the crucial factors in integrating global competence into English teaching and learning and, as a result, identifying appropriate actions to assist ESL teachers’ global competence development through programs, workshops, and other suitable platforms.

### Research question

Thus, the purposes of this study are to address the perceptions and opinions of ESL teachers on global competence in English lessons and identify the factors that affect the integration of global competence in ESL lessons. This study seeks to answer the following research questions:

1. What are the ESL teachers’ perceptions of teachers’ global competence in English teaching and learning?
2. What are the ESL teachers’ perceptions of professional development programs organized by the MOE in developing their global competence?

## Methodology

### Research design

This study employed a quantitative method that identified ESL teachers’ perceptions and factors regarding global competence in English teaching and learning. Due to the objectives of this study, this study used a cross-sectional survey design in which the researcher collected data from a questionnaire distributed to respondents. This study provides a primary overview of an actual study on a larger scale.

### Research sample

This study applied a purposive sampling method, seeking responses from the respondents who fit the list of criteria. A total of 172 Malaysian English teachers teaching secondary school students aged between 13 and 18 were the respondents for this study. The criteria for the respondents were: (1) L2 teachers, (2) teaching in public secondary schools, (3) teaching in Malaysia, and (4) having experience attending professional development programs/workshops/courses. For selecting respondents who corresponded to the fourth criterion, the ESL teachers made their judgments based on the descriptions of the criteria included with the Google Form Survey link. The requests for participation in the survey, the descriptions, and links were sent to the ESL teachers *via* online platforms.

The data collection was conducted between April 5 and April 18, 2021. The teachers who fit the criteria voluntarily agreed to respond to the survey questionnaire during their free time within the period allocated by the researcher in a fortnight.

### Research instrument

This research utilized the quantitative research design to identify the perceptions of ESL teachers in Malaysia on global competence. The survey questionnaire was designed to extract information on the perceptions of ESL teachers. The construction referred to the study’s objectives and the research questions formulated. Through surveys, information from many respondents can be obtained and further analyzed.

Before conducting the study, a pre-test was performed to confirm no ambiguity in each questionnaire item and that the respondents could clearly understand the questions (Memon et al., 2017). Five respondents were selected to read the items and fill out the questionnaire in performing the pre-test. It is to verify the clarity of the items as to whether the target sample would understand the questions and respond appropriately (Memon et al., 2017). Since the instrument was not too long, this study followed the recommendation to use 5 to 15 samples (Beatty and Willis, 2007) for the pre-test. The pre-testing provided results on whether:

1. Correct wording for the items.
2. Correct sequence of the items.
3. Clearly understood items (by the respondents).
4. Additional items or exclusion of items.
5. Clear and adequate instructions.

Necessary amendments were made to improve the items based on feedback from five respondents. Then, the web-based survey questionnaire was submitted for expert checking to further validate the survey items before distributing them for the actual data collection. It is summarized that all the items and the data collected from the survey were reliable. Besides, the web-based survey was appropriate for the safety guidelines of reducing physical contact and heightening physical distancing due to the recent Coronavirus or COVID-19 pandemic. It was also helpful to expedite and improve the process (Creswell and Creswell, 2018). In this study, the 5-point Likert scale items with the scale ranging from 1: Strongly Disagree (SA), 2: Disagree (D), 3: Quite Agree (QA), 4: Agree (A), and 5: Strongly Agree (SA) were used. Furthermore, the quantitative analysis from the survey provided a demographic description of the respondents and the frequencies of multiple responses received for each item.

### Data collection procedure and analysis

A web-based survey link or Google Form was given to ESL teachers and posted in English teachers’ groups through social media platforms and messaging applications such as Telegram, WhatsApp, Facebook, Twitter, and Instagram. It was the fastest and most efficient way to reach the teachers in different states.

The online data gathered from the Google Form questionnaire were tabulated. The responses for each item were entered into an IBM SPSS Statistics database version 26 for the descriptive quantitative analysis procedure. The descriptive analysis described a particular situation through numerical data (Mat Roni et al., 2020), and all the data were analyzed using frequency and percentage. The mean scores were then rated, referring to the agreement level in Table 1 below.

**Table 1:** Five-point Likert scale agreement level (Adapted from Altan, 2018).

Rating	Mean	Agreement Level
5	4.51–5.00	Very high
4	3.51–4.50	High
3	2.51–3.50	Moderate
2	1.51–2.50	Low
1	1.00–1.50	Very low

## Results

The data collected from the online survey questionnaire are presented and discussed in two sections, categorized below, with the first section on ESL teachers’ knowledge and perceptions of global competence in English lessons. Finally, the second section elaborated on the ESL teachers’ perceptions of professional development programs organized by the MOE to develop their global competence.

Table 2 displays the demographic data. One hundred and forty-three female ESL teachers (83.1%) and 29 male ESL teachers (16.9%) teaching English in secondary schools all over Malaysia participated in this study. The majority of the respondents were between 31 to 40 years old, with 67 teachers (39%). Besides, all the respondents had different years of experience teaching English, with the lowest percentage of ESL teachers teaching between 1 and 5 years (8.1%). The highest response was from ESL teachers with more than 16 years of experience (47.1%).

**Table 2:** Demographic background.

Item	Category	Percentage
1. Gender	Male	83.1
	Female	16.9
2. Age	25–30 years	11
	31–40 years	39
	41–50 years	29.1
	51 years old and above	20.9
3. State	Melaka	15.7
	Johor	18.6
	Negeri Sembilan	5.2
	Kuala Lumpur/Selangor/W.P./Putrajaya	8.7
	Kedah	9.3
	Kelantan	2.9
	Pahang	5.8
	Penang	2.3
	Perak	7.6
	Perlis	0.6
	Terengganu	9.3
	Sabah	8.7
	Sarawak	5.2
4. School	Urban	32
	Semi-urban	39.5
	Rural	32
5. Years of Experience in Teaching English	1–5 years	8.1
	6–10 years	27.9
	11–15 years	16.9
	16 years and above	47.1
6. Have you attended 10 or more courses/programs/workshops on developing global competence	Yes	83.1
	No	16.9

Next, the respondents’ criteria in this study include the experience of attending professional development programs. In the questionnaire employed, one of the items is specific to gather responses on whether the ESL teachers have previously attended 10 or more programs on global competence. Regarding Item 6, 143 ESL teachers (83.1%) answered “Yes,” while the other 29 ESL teachers (16.9%) said “No.”

### ESL teachers’ knowledge and perceptions on global competence

In this section, 14 items measured the respondents’ knowledge and perceptions of global competence to answer this study’s first research question (RQ1). The results are presented in Table 3:

**Table 3:** Percentages and mean scores of teachers’ perceptions.

Items	Strongly disagree 1	Disagree 2	Quite agree 3	Agree 4	Strongly agree 5	Mean score
1. I know the definition of global competence.	6 (3.5%)	20 (11.6%)	67 (39%)	59 (34.3%)	20 (11.6%)	3.39
2. I know the elements of global competence.	13 (7.6%)	28 (16.4%)	69 (40.4%)	51 (29.8%)	10 (5.8%)	3.10
3. I consider myself as a globally competent ESL teacher.	5 (2.9%)	14 (8.1%)	89 (51.7%)	57 (33.1%)	7 (4.1%)	3.27
4. I have the characteristics of a globally competent ESL teacher.	4 (2.3%)	15 (8.7%)	84 (48.8%)	58 (33.7%)	11 (6.4%)	3.33
5. I know that intercultural competence is different than global competence.	6 (3.5%)	19 (11%)	68 (39.5%)	62 (36%)	17 (9.9%)	3.38
6. I believe that global competence is closely-related to English (ESL) lessons.	4 (2.3%)	17 (9.9%)	42 (24.4%)	71 (41.3%)	38 (22.1%)	3.71
7. ESL teachers’ global competence is important.	0 (0%)	1 (0.6%)	20 (11.6%)	83 (48.3%)	68 (39.5%)	4.27
8. I have a positive view on the integration of global competence in ESL lessons.	0 (0%)	1 (0.6%)	23 (13.4%)	84 (48.8%)	64 (37.2%)	4.23
9. Integrating global competence in ESL lessons is important.	0 (0%)	0 (0%)	25 (14.6%)	87 (50.9%)	59 (34.5%)	4.20
10. Integrating global competence creates meaningful ESL lessons.	0 (0%)	3 (1.7%)	26 (15.1%)	82 (47.7%)	61 (35.5%)	4.17
11. Integrating global competence assists ESL students’ English learning.	0 (0%)	1 (0.6%)	30 (17.4%)	80 (46.5%)	61 (39.5%)	4.17
12. I agree that students engage better when ESL teachers teach global knowledge.	0 (0%)	2 (1.2%)	40 (23.3%)	78 (45.3%)	52 (30.2%)	4.05
13. I believe that students become more interested when ESL teachers teach global knowledge.	0 (0%)	2 (1.2%)	35 (20.3%)	77 (44.8%)	058 (33.7%)	4.11
14. ESL lessons should include knowledge of global and local issues.	0 (0%)	2 (1.2%)	15 (8.8%)	74 (43.3%)	80 (46.8%)	4.35

As depicted in Tables 3, a moderate item-total mean was indicated in the ESL teachers’ consideration of themselves as globally competent teachers (*M* = 3.27) and the perceptions that they have the characteristics of globally competent ESL teachers (*M* = 3.33). Similarly, a moderate level of agreement was found in the ESL teachers’ knowledge of the global competence definition (*M* = 3.39) and elements (*M* = 3.10). Another moderate agreement was found among some ESL teachers regarding their knowledge of the difference between intercultural and global competence (*M* = 3.38).

The respondents were asked if they believed that global competence is closely related to English lessons. Based on the high mean score of *M* = 3.71, the majority agreed. Interestingly, most of them understood the importance of global competence in teaching English. The ESL teachers mostly agreed (*M* = 4.27) with the notion that ESL teachers’ global competence is essential. Furthermore, the high level of agreement (*M* = 4.23) on the ESL teachers’ view of global competence integration in English lessons showed that most ESL teachers strongly agreed and agreed that they have a positive and favorable view of the integration. Most ESL teachers strongly felt that integrating global competence into lessons is crucial (*M* = 4.20). Most respondents also agreed that the integration creates meaningful lessons and assists students’ English learning (*M* = 4.17).

Moreover, based on the high level of agreement for Items 12 and 13, most ESL teachers noticed that students displayed better engagement (*M* = 4.05) and interest (*M* = 4.11) in learning the target language when global competence was integrated into language lessons. From the findings, most ESL teachers agreed that English lessons should emphasize knowledge of local and global issues (*M* = 4.35); with 8.8% (15) ESL teachers quite agreed, 43.3% (74) agreed, and 46.8% (80) strongly agreed. The strong agreement among the respondents concerning such content to be imparted in ESL lessons showed their perceptions about current ESL teaching and learning. Most were also optimistic about the global competence integration in ESL lessons.

### ESL teachers’ perceptions on professional development programs by the ministry of education (MoE) in developing ESL teachers’ global competence

The data gathered from the survey concerning ESL teachers’ perceptions of the particular programs by MoE were analyzed for the mean scores. Table 4 below displays the findings generated from the descriptive statistic test:

**Table 4:** Percentages and mean scores of the professional development programs by the Ministry of Education for ESL teachers’ global competence development.

Items	Strongly disagree 1	Disagree 2	Quite agree 3	Agree 4	Strongly agree 5	Mean score
1. The CEFR-aligned syllabus offers a platform/medium for ESL teachers to integrate global competence in ESL lessons.	0 (0%)	0 (0%)	27 (15.8%)	77 (45%)	67 (39.2%)	4.23
2. The CEFR-aligned courses provide exposure for ESL teachers on how to inculcate global competence in English lessons.	0 (0%)	0 (3.5%)	29 (16.9%)	73 (42.4%)	64 (37.2%)	4.13
3. The professional development courses attended improve my global competence (as an ESL teacher).	5 (2.9%)	11 (6.4%)	55 (32%)	74 (43%)	27 (15.7%)	3.62
4. I gain knowledge about global issues from courses organized by MOE.	11 (6.4%)	31 (18%)	58 (33.7%)	53 (30.8%)	19 (11%)	3.22
5. MOE has helped me (as an ESL teacher) in the development of my global competence.	12 (7%)	33 (19.2%)	60 (34.9%)	50 (29.1%)	17 (9.9%)	3.16
6. Materials regarding global issues are easily accessible offline and online.	0 (0%)	6 (3.5%)	29 (16.9%)	72 (41.9%)	65 (37.8%)	4.14

The quantitative data findings provided information regarding ESL teachers’ perceptions regarding the factors for the second research question (RQ2). The survey indicated how the programs and courses by the MOE might affect ESL teachers’ global competence development. A high mean score revealed some of the respondents’ high level of agreement that the CEFR-aligned syllabus and related courses offer platforms for teachers to integrate global competence (*M* = 4.23). According to the results, the high score implied that some ESL teachers were keen to learn and develop global competence through various programs. The vital role of programs and courses for ESL teachers’ professional development could not be denied. Based on Table 4, the high agreement among some respondents on Item 3 indicated some ESL teachers’ belief that the professional development courses have improved their global competence (*M* = 3.62). However, the data analysis also resulted in a moderate level of agreement on whether the ESL teachers have received knowledge on global issues from courses organized by MOE (*M* = 3.22). The disparity in ESL teachers’ perceptions about the courses organized by MOE (*M* = 3.22) showed the scarcity of suitable programs that cater to ESL teachers’ global competence needs.

Global competence knowledge must be embedded in offered courses so that teachers know the interdisciplinary connections of global competence development. The lowest mean score for whether the MOE has helped the ESL teacher develop global competence (*M* = 3.16) proves the lack of help from MOE to train globally competent ESL teachers. The respondents had different opinions about it, and 34.9% quite agreed with the claim, 19.2% disagreed, and 7% strongly disagreed. The mixed reactions to the content of Item 5 may require the MOE’s attention on how to better assist ESL teachers in developing their global competence.

In this study, some of the respondents believed that the availability of materials on global issues had helped them (*M* = 4.14) widen their content for the ESL lessons. Generally, it boosts their confidence in teaching global knowledge in language classrooms. The ESL teachers’ high level of agreement on Item 6 reflects their ability to find materials on global issues independently.

It can be concluded that ESL teachers generally have positive perceptions toward integrating global competence into English teaching and learning. Most were aware of global competence and its benefits to their students’ ESL learning. Therefore, professional development programs that aim to develop ESL teachers’ global competence and globally competent teaching are needed to strengthen their knowledge, skills, and dispositions toward global competence.

## Discussion

Overall, the study revealed that Malaysian ESL teachers were keen on integrating global competence in English lessons. From the findings, this study uncovers most ESL teachers’ positive perceptions about their global competence and the integration of global competence into English teaching. Most ESL teachers believed that global competence is significant for teachers and students. It is in line with Tichnor-Wagner et al. (2019) that said teachers need to develop global competence to assist students’ global competence development. Global competence is crucial as it enhances numerous skills such as effective communication, logical reasoning, intercultural adeptness, conflict resolution, perspective taking, and adaptability (Kerkhoff et al., 2019; Sinagatullin, 2019; Stankovska et al., 2019). As a result, ESL students will learn the target language more meaningfully.

The moderate mean scores on the ESL teachers’ knowledge and understanding of global competence may indicate the lack of knowledge of ESL teachers on the definition and elements of global competence. A possible explanation is that some of them were aware of global competence but did not recognize all global competence elements as listed by scholars. Some may know the meaning of global competence but not to the extent of considering themselves globally competent ESL teachers. Interestingly, the results showed a contrasting view from the high number of ESL teachers who agreed to have attended 10 or more global competence-related programs. The programs might be lacking in effectively and specifically imparting knowledge of global competence for the teachers’ development. Thus, providing specific support, guidance, courses, and professional development programs is necessary to enhance ESL teachers’ understanding of global competence. As suggested by Ukpokodu (2020), enough support will assist ESL teachers in teaching students’ similar knowledge, skills, and dispositions of global competence necessary for the ESL students’ future.

The high level of agreement signified the positive perceptions of the respondents regarding the application of globally competent ESL teaching. Contrary to their specific knowledge of global competence, the findings showed that most ESL teachers agreed on the significance of global competence in ESL lessons. ESL teachers who incorporate global competence in lessons are considered globally competent and practiced globally competent teaching. A study by Tamerat (2020) shared a similar viewpoint that most teachers described global competence with multiple definitions of global education and were influenced by the elements that were personally relevant to their understanding. The author explained that the respondents in the study had interchanged concepts, including global and cultural competence.

Additionally, the findings revealed the ESL teachers’ perceptions of professional development programs by the Ministry of Education in Malaysia. Based on the data analysis, it can be assumed that most ESL teachers believed that professional development programs are needed to improve their global competence. It is due to the high agreement level to their global competence improvement through programs. However, a contrasting perspective on whether the MOE programs have enhanced ESL teachers’ knowledge of global issues showed that most ESL teachers lamented the scarcity of specific programs by MOE. A possible explanation is that the MOE has not explicitly organized programs to prepare ESL teachers for globally competent teaching. Tichnor-Wagner et al. (2019) and Ukpokodu (2020), in their respective studies, have also found that the programs organized by the authorities or government education departments in their respective countries lacked the specifications to develop teachers’ global competence and globally competent teaching. Generally, continuous professional development programs could hone teachers’ teaching skills and keep them updated with the relevant teaching content (Vadivel et al., 2021). Thus, it is vital for stakeholders and education departments worldwide to create specific programs that are systemically structured to develop global competence among teachers.

Overall, the findings in this study questioned the MOE efforts in developing Malaysian English teachers’ global competence in teaching. The moderate agreement level showed that most ESL teachers doubted the role of MOE in assisting their global competence development. As previously reported, some programs organized by MOE did not achieve their objective based on the discouraging English teachers’ participation and the organizers’ lack of effort to consult teachers and design programs that suit their needs (Sukri et al., 2017). The authors further stated that insufficient programs are conducted to cater to teachers’ global awareness needs. Therefore, this study may provide strong pedagogical implications for English teachers, curriculum and syllabus designers, and ESL teacher education program designers to design suitable programs according to the practitioners’ current needs.

The education programs should be reviewed to provide more inclusive and impactful training, such as strengthening their understanding of global competence element. Chu et al. (2021) supported that “teacher qualities are situated in the setting where teachers positively interact with the surrounding environments” (p: 3). ESL teachers will benefit more from understanding methods to teach worldwide issues, managing stereotypes, multicultural issues, and conflicts. Abdullah and Abdullah (2018) and Chong and Yamat (2021) agreed that critical issues, including managing stereotypes, biases, and discriminatory behavior among students, need to be addressed in ESL education due to the inclusion of global topics in the CEFR-aligned syllabus. Understanding the topics in-depth and addressing more local and global issues in ESL lessons are crucial.

## Conclusion

In general, it can be concluded that ESL teachers’ understanding of the importance of global competence was relatively high. ESL teachers need global competence to help meet the challenges inherent in educating students to be linguistically and globally competent in today’s interconnected world. Most teachers emphasized the feasibility of presenting global knowledge and issues to students at any level of English proficiency. This study offers insights into global competence awareness and globally competent teaching among ESL teachers. The data also revealed that most ESL teachers perceived their role in engaging students with local and global content to learn English. Thus, this study has emphasized the need to situate global consciousness in teaching English. The interconnected and interdependent world requires ESL teachers to teach language beyond the linguistics paradigm across the worldwide content and cultures of the language.

From the findings in this study, the MOE can gain insights into creating future programs specific to ESL teachers’ global competence development. ESL teachers’ professional development is recommended to be intimately connected to global competence and globally competent teaching. They should be trained with critical skills to educate students on their roles and responsibilities. Besides, professional learning communities in schools and ESL teachers nationwide are vital to narrowing the global knowledge gap. Positive agreement among the ESL teachers to achieve globally competent teaching will create a community of active educators.

The results obtained are valuable for future research in the same interests in the Malaysian context. The present study summarizes that global competence among ESL teachers is essential, and specific global competence development programs should be systematically organized. This study’s limitation is that the impacts of English teachers’ global competence and globally competent teaching on students’ learning are not identified. Future research could consider looking into these subject matters more profoundly and in a large-scale study. It is even more valuable for future research to use various research designs that could show more intensity in examining ESL teachers’ opinions. In addition, future studies on global competence may compare the perceptions of experienced and novice English teachers.

## Author’s note

NSY is a teacher in Malaysia and a doctorate student taking TESL at the National University of Malaysia. She has previously published papers and is interested in the use of technology in ESL classrooms, global competence in teaching and learning, teachers’ professional development as well as teaching and learning pedagogy. MMY is a Professor and Deputy Dean of Research and Innovation at the Faculty of Education, National University of Malaysia. She is best known for establishing the integration of ICT in teaching and learning English. She is active in scholarly journal writing and publishing and has published papers in various Citation-Indexed journals. HH is a senior lecturer and an assistant professor at the Centre for Teaching and Learning Innovations, Faculty of Education, Universiti Kebangsaan Malaysia (UKM). She is an educational technology enthusiast and an e-learning practitioner. Her areas of concentration are ESL, mobile learning, Mobile-assisted Language Learning (MALL), technology acceptance as well as language pedagogy, and the use of technology in teaching English as a Second Language (ESL).

## Data availability statement

The original contributions presented in the study are included in the article/supplementary material, further inquiries can be directed to the corresponding author.

## Author contributions

All authors listed have made a substantial, direct, and intellectual contribution to the work and approved it for publication.

## Funding

Funds received for open access publication fees, from my institution (grants), National University of Malaysia/ Universiti Kebangsaan Malaysia with grant no. GG-2020-024.

## Conflict of interest

The authors declare that the research was conducted in the absence of any commercial or financial relationships that could be construed as a potential conflict of interest.

## Publisher’s note

All claims expressed in this article are solely those of the authors and do not necessarily represent those of their affiliated organizations, or those of the publisher, the editors and the reviewers. Any product that may be evaluated in this article, or claim that may be made by its manufacturer, is not guaranteed or endorsed by the publisher.
